# MR spectroscopy using static higher order shimming with dynamic linear terms (HOS‐DLT) for improved water suppression, interleaved MRS‐fMRI, and navigator‐based motion correction at 7T

**DOI:** 10.1002/mrm.28202

**Published:** 2020-02-14

**Authors:** Vincent O. Boer, Mads Andersen, Anna Lind, Nam Gyun Lee, Anouk Marsman, Esben T. Petersen

**Affiliations:** ^1^ Danish Research Centre for Magnetic Resonance Centre for Functional and Diagnostic Imaging and Research Copenhagen University Hospital Hvidovre Denmark; ^2^ Philips Healthcare Copenhagen Denmark; ^3^ Department of Biomedical Engineering University of Southern California Los Angeles California; ^4^ Center for Magnetic Resonance Department of Health Technology Technical University of Denmark Kgs. Lyngby Denmark

**Keywords:** ^1^H MR spectroscopy, B_0_ shimming, EPI distortions, prospective motion correction, ultra‐high field, water suppression

## Abstract

**Purpose:**

To interleave global and local higher order shimming for single voxel MRS. Single voxel MR spectroscopy requires optimization of the B_0_ field homogeneity in the region of the voxel to obtain a narrow linewidth and provide high data quality. However, the optimization of local higher order fields on a localized MRS voxel typically leads to large field offsets outside that volume. This compromises interleaved MR sequence elements that benefit from global field homogeneity such as water suppression, interleaved MRS‐fMRI, and MR motion correction.

**Methods:**

A shimming algorithm was developed to optimize the MRS voxel homogeneity and the whole brain homogeneity for interleaved sequence elements, using static higher order shims and dynamic linear terms (HOS‐DLT). Shimming performance was evaluated using 6 brain regions and 10 subjects. Furthermore, the benefits of HOS‐DLT was demonstrated for water suppression, MRS‐fMRI, and motion corrected MRS using fat‐navigators.

**Results:**

The HOS‐DLT algorithm was shown to improve the whole brain homogeneity compared to an MRS voxel‐based shim, without compromising the MRS voxel homogeneity. Improved water suppression over the brain, reduced image distortions in MRS‐fMRI, and improved quality of motion navigators were demonstrated using the HOS‐DLT method.

**Conclusion:**

HOS‐DLT shimming allowed for both local and global field homogeneity, providing excellent MR spectroscopy data quality, as well as good field homogeneity for interleaved sequence elements, even without the need for dynamic higher order shimming capabilities.

## 1 INTRODUCTION

^1^H‐magnetic resonance spectroscopy (MRS) is a powerful tool for the noninvasive detection of biochemical processes in the brain. Both the signal‐to‐noise ratio (SNR) and chemical shift dispersion increase with field strength, hence ultra‐high field MRS can provide improved detection sensitivity for numerous metabolites.^1^ It is important to note that the quantification accuracy of these metabolites is a complex function of spectral characteristics. One of the important factors is the overlap with other metabolites, which decreases with higher field.^2^ However, in order to benefit from the increases in SNR and spectral resolution at higher field, it is essential that the local field homogeneity in the MRS voxel is optimized by the use of higher order shimming.^1^ At field strengths up to 3T, the field homogeneity in the voxel can typically be improved enough by using linear gradient fields. But at and above 3T, higher order shim (HOS) coils are typically used, which provide spherical harmonic field shapes of higher degree and order to counter the increased field distortions induced by the human body at higher field.^3^ Although this provides good B_0_ field homogeneity in the voxel, using higher order fields can lead to undesired high field offsets outside the voxel.^4^, ^5^ This compromises the use of sequence elements that perform best with good global field homogeneity such as slice selective RF pulses used for voxel selection and outer volume suppression.

One more example of such a sequence element is narrow‐band water suppression in ^1^H MRS. A poor field homogeneity over the brain compromises global water suppression values and increases the chances of spurious echoes generated outside the voxel.^6^

Another example is interleaved MRS and functional MRI (fMRI). Combined spectroscopy and functional imaging studies show great potential to simultaneously acquire neurochemical and hemodynamic measures.^7^ This, however, also requires good field homogeneity in the MRS voxel, as well as full brain homogeneity for whole brain fMRI, as fMRI is typically performed using gradient‐echo echo planar imaging (GE‐EPI). GE‐EPI sequences typically have a low bandwidth in the phase encoding direction, which can lead to severe distortions in regions with poor field homogeneity as well as signal dropouts due to the lack of a refocusing pulse.

Similarly, global field homogeneity is important when performing motion corrected MRS using interleaved MR‐based navigators. Although motion correction is possible using external tracking devices,^8^, ^9^, ^10^ MR navigators^11^ have the advantage that they are inherently acquired in the scanner coordinate system. Also they do not require additional hardware or the use of markers, while still showing a significant improvement in clinical scoring of motion corrupted scans.^12^ Fat‐navigators^13^ allow for parallel imaging acceleration at high accuracy^14^ and minimal interaction between the MRS and navigator signal. However, the navigators require good whole brain field homogeneity to ensure fat selectivity everywhere, whereas MRS requires an excellent local field homogeneity in the MRS voxel.

To accomplish good homogeneity for a small target region for MRS and whole brain homogeneity for the aforementioned interleaved sequence elements, an optimal solution can be found in dynamic shimming,^3^ where separate shim sets are calculated for the MRS voxel and the interleaved sequence element. This is similar to the idea of having a separate shim set for every slice in a multislice sequence,^15^ but then using an update between the MRS localization and acquisition, and for example, the water suppression.^16^ By switching between these shim sets during scanning, both global and local field homogeneity can be achieved when required. At 3T, for example, motion corrected MRS was performed with separate linear shim sets for the navigator and MRS voxel, which were switched dynamically.^11^ The gradient coils in modern human MRI systems are designed for rapid switching, as they are shielded, and preemphasis is performed per default. However, this is generally not the case for higher order shim coils, and higher order dynamic shimming is thus complicated by severe eddy currents that are induced by switching the shim currents. Typical time constants are in the order of seconds,^17^ which is too long for practical use without any eddy current compensation. Full high order dynamic shimming thus requires a complex calibration procedure for eddy current preemphasis and cross‐term compensation^17^, ^18^ and/or additional hardware^19^ to drive the shims. Even then, the dynamic range of the shims is reduced significantly to allow for eddy current compensation, restricting shim performance.^20^ Additional regularization can be employed to minimize switched eddy currents^21^ and there is research into the use of alternative shim systems that do not induce large eddy currents and can be used in a dynamic way.^22^

Here we investigate the use of an optimized shimming algorithm for interleaved local and global shimming, without switching the higher order shim fields. As the higher order shim terms are typically not fully orthogonal in the shim volume, it has been observed that the numerical optimization has a solutions space where multiple solutions provide similar B_0_ shimming quality.^4^, ^5^ Here we propose to exploit this property of higher order shimming to use a single step cost‐function minimization of the static higher order shims while allowing dynamically adapting linear shims (HOS‐DLT).

We evaluated the HOS‐DLT method by shim simulations on B_0_ field map data with MRS in different brain regions. Furthermore, we show three applications of interleaved local and global shimming with HOS‐DLT. First, improvement of water suppression in single voxel (SV) MRS with narrow‐band water suppression is shown. Second, reduced image distortions of GE‐EPI images from interleaved MRS‐fMRI are shown. Third, the use of HOS‐DLT in MRS with prospective motion correction using fat‐navigators is shown, enabling the use of HOS for ultra‐high field motion correction.

## 2 METHODS

### 2.1 Shimming algorithms

Three different approaches to B_0_ field shimming were considered and described below. In all cases, B_0_ field shimming was approached as the constrained minimization problem of finding the shim currents within the amplifier limits, which maximizes the homogeneity of the B_0_ magnetic field.

### 2.2 Single volume of interest shimming (MRS shim VOI/brain shim VOI)

The most straightforward approach to shimming is the least‐squares minimization of the B_0_ field in a single volume of interest (VOI)

(1) $$\begin{array}{c} \underset{s}{\text{minimize}\;}\;{∥b_{0}\;-\;As∥}_{2}^{2}\\ \text{subject to}\;-s_{\mathrm{limit}}\;\leq \;s\left[i\right]\;\leq \;s_{\mathrm{limit}},\;i=1,\ldots ,\;N \end{array}$$

where $b_{0}\;\in \;R^{M}$ is the measured B_0_ field before shimming in the MRS shim VOI or brain shim VOI (in Hz). The matrix $A\in R^{\mathrm{MxN}}$ contains the concatenated spatial shim field basis functions that the shim coils produce for the measurement points in the VOI (in Hz/A). The vector $s\;\in \;R^{N}$ contains the shim current amplitudes (in A) for the different shim coils. For the MRS shim VOI, a volume slightly larger than the MRS voxel was used (extended 10 mm in each direction) to reduce sensitivity to subject motion. For shimming on the whole brain, a brain extraction algorithm was used^23^ to define the brain shim VOI.

### 2.3 Dynamic shimming

For sequences requiring both local and global homogeneity, both the MRS shim and brain shim can be used in an interleaved way. For dynamic linear shimming this does not pose any restraints as induced eddy currents from switching linear shims after eddy current compensation are typically small. Therefore, dynamic linear shimming can be used relatively easily. However, for higher order dynamic shimming, the residual eddy currents generated by the higher order shim coil switching can be in the order of seconds and would deteriorate the spectral quality. Therefore, higher order eddy current compensation should be performed. This is, however, not widely available and requires additional hardware and calibration^3^ and compromises the maximum shim currents that can be used.^20^ This analysis was used (ignoring the reduced maximum shim constraints) to estimate the theoretical best possible simultaneous shim outcome for both the voxel and brain.

### 2.4 Weighted static higher order shimming (wHOS shim)

To achieve good homogeneity in two VOIs simultaneously, a cost‐function was used for a weighted optimization between the MRS shim and the whole brain shim.^4^, ^5^

(2) $$\begin{array}{c} \underset{s}{\text{minimize}}\;{∥b_{0,v}\;-\;A_{v}s∥}_{2}^{2}\;+\;\alpha {∥b_{0,b}\;-\;A_{b}s∥}_{2}^{2}\\ \text{subject to}\;-s_{\mathrm{limit}}\;\leq \;s\left[i\right]\;\leq \;s_{\mathrm{limit}},\;i=1,\ldots ,\;N \end{array}$$

where $b_{0,v}\;\in \;R^{M}$ is the measured B_0_ field in the MRS shim VOI, $b_{0,b}\;\in \;R^{P}$ is the measured B_0_ field in the brain shim VOI, and the matrices $A_{v}\;\in \;R^{\mathrm{MxN}}$ and $A_{b}\;\in \;R^{\mathrm{PxN}}$ contain the shim field basis functions for the MRS shim VOI and the brain shim VOI, respectively. The vector $s\;\in \;R^{N}$ contains the shim current amplitudes. This optimization problem can be rewritten as

(3) $$\begin{array}{c} \underset{s}{\text{minimize}}{∥\left[\begin{array}{c} b_{0,v}\\ \sqrt{\alpha }b_{0,b} \end{array}\right]-\left[\begin{array}{c} A_{v}\\ \sqrt{\alpha }A_{b} \end{array}\right]s∥}_{2}^{2}\\ \text{subject to}\;-s_{\mathrm{limit}}\;\leq \;s\left[i\right]\;\leq \;s_{\mathrm{limit}},\;i=1,\ldots ,\;N \end{array}$$

to yield a computationally efficient matrix formulation that is convex and can be efficiently solved to find the global minimum.

### 2.5 Weighted static higher order shimming with dynamic linear terms (HOS‐DLT shim)

The weighted optimization was extended using separate sets of linear shims for the VOIs, resulting in two vectors with shim currents *s_v_* and *s_b_* for the MRS shim and whole brain shim respectively with shared higher order shim currents but different linear shim currents

(4) $$\begin{array}{c} \underset{s_{v},s_{b}}{\text{minimize}}{∥b_{0,v}\;-\;A_{v}s_{v}∥}_{2}^{2}\;+\;\alpha {∥b_{0,b}\;-\;A_{b}s_{b}∥}_{2}^{2}\\ \text{subject to}\;-s_{\mathrm{limit}}\;\leq \;s_{v}\left[i\right],\;s_{b}\left[i\right]\;\leq \;s_{\mathrm{limit}},\;i=1,\ldots ,\;N, \end{array}$$

where $s_{v}={\left[s_{v,L},\;s_{H}\right]}^{T}\in R^{N}$ and $s_{b}={\left[s_{b,L},\;s_{H}\right]}^{T}\in R^{N}$. The vectors $s_{v,L}\;\in \;R^{4}$ and $s_{b,L}\;\in \;R^{4}$, herein denoted dynamic linear terms (DLT), consist of the zeroth component and shim currents for the x, y, and z gradient coils in the MRS shim VOI and brain shim VOI, respectively. The vector $s_{H}\;\in \;R^{N-4}$ contains the shared static higher order shim currents.

The $A_{v}$ and $A_{b}$ matrices can be split into a linear ( $A_{v,L},\;A_{b,L})$ and higher order part ( $A_{v,H},\;A_{b,H})$ in order to rewrite the minimization problem in matrix form:

(5) $$\begin{array}{c} \underset{s_{v,L},s_{b,L},s_{H}}{\text{minimize}}{∥\left[\begin{array}{c} b_{0,v}\\ \sqrt{\alpha }b_{0,b} \end{array}\right]-\left[\begin{array}{ccc} A_{v,L} & 0 & A_{v,H}\\ 0 & \sqrt{\alpha }A_{b,L} & \sqrt{\alpha }A_{b,H} \end{array}\right]\left[\begin{array}{c} s_{v,L}\\ s_{b,L}\\ s_{H} \end{array}\right]∥}_{2}^{2}\\ \text{subject to}\;-s_{\mathrm{limit}}\;\leq \;s_{v,L}\left[i\right],\;s_{b,L}\left[i\right]\;\leq \;s_{\mathrm{limit}},\;i=1,\ldots ,\;4\\ -s_{\mathrm{limit}}\;\leq \;s_{H}\left[i\right]\;\leq \;s_{\mathrm{limit}},\;i=N-4,\ldots ,\;N. \end{array}$$

For all three shimming algorithms, the minimization problem was solved with a linear constrained optimizer in Matlab (The Mathworks Inc., Natick, Massachusetts).^24^, ^25^

### 2.6 Measurements

All human experiments were performed in accordance to the local ethical guidelines and written informed consent was obtained from all participants. Data was acquired with a volume transmit coil and a 32‐channel receiver array (Nova Medical Inc., Burlington, Massachusetts) using a whole body 7T MRI system (Achieva, Philips Healthcare, Best, The Netherlands) equipped with a full third order spherical harmonic shim system. Each higher order shim coil was fed with a +/− 10 A current amplifier. In all experiments a B_0_ field map was acquired for optimizing the shim settings, as well as a T_1_‐weighted anatomical scan (MP‐RAGE with isotropic voxel size of 0.8 mm^3^) for positioning of the spectroscopy voxels. The B_0_ field map was a 3D gradient echo sequence with field of view (FOV) of 240 × 240 × 116 mm^3^, isotropic voxel size of 3.75 mm^3^, echo time TE1/TE2/repetition time (TR) = 2/3/10 ms.

Single voxel MRS data was acquired with a semi‐localization by adiabatic‐selective refocusing (sLASER)^26^ sequence employing VAPOR water suppression,^27^ TE/TR = 32/3600 ms, number of averages of 16 with 1 non‐water suppressed acquisition for coil addition and eddy current correction.

### 2.7 Field simulations in a group of subjects

An optimal weighting value, α, for both the wHOS shim and the HOS‐DLT shim was determined on B_0_ map data from one subject with an MRS voxel in the hippocampus. The dependence of the standard deviation of the field in both the MRS VOI and brain VOI on the weighting value α were assessed, and the value for α was determined at a threshold of 0.5 Hz above the optimal MRS shim using third order shimming. Although the optimization to find this value can be run for every subject and voxel location this would require some significant calculation time. We chose to use the α determined using the hippocampus voxel location in one volunteer for the following experiments in this work.

The performance of the MRS shim, the whole brain shim and HOS‐DLT was assessed on B_0_ maps from 10 subjects with manually planned voxels in 6 brain regions each; the anterior cingulate cortex (20 × 20 × 20 mm^3^), the motor cortex (20 × 20 × 20 mm^3^), the dorsolateral prefrontal cortex (20 × 20 × 20 mm^3^), the supraventricular white matter (20 × 20 × 20 mm^3^), the hippocampus (30 × 15 × 15 mm^3^) and the thalamus (16 × 12 × 16 mm^3^).

### 2.8 Residual water suppression imaging

An MRS sequence was interleaved with a gradient echo imaging sequence using an interleaved scanning framework.^28^ The MRS localization and acquisition were omitted, but the water suppression was still performed using the VAPOR sequence^27^ with a bandwidth of 200 Hz. By switching over to the imaging sequence right after the end of the VAPOR sequence, the water suppression efficiency could be imaged.^29^ The gradient echo sequence acquired a single slice with thickness of 4 mm, in‐plane voxel size was 1.5 × 1.5 mm^2^, TE/TR = 1.5/10 ms, and k‐space filling order was from center and outwards. The water suppression ratio was calculated from images acquired with and without water suppression. The experiment was performed using both the MRS shim and HOS‐DLT shim with the MRS shim VOI in the occipital lobe to assess the extent of the water suppression using the different shim approaches.

Single voxel sLASER MRS data were acquired from the occipital cortex using both the MRS shim and HOS‐DLT shim to assess the level of artificial stimulated echoes caused by improved water suppression over the brain.

### 2.9 Interleaved MRS‐fMRI

**Figure 1:**
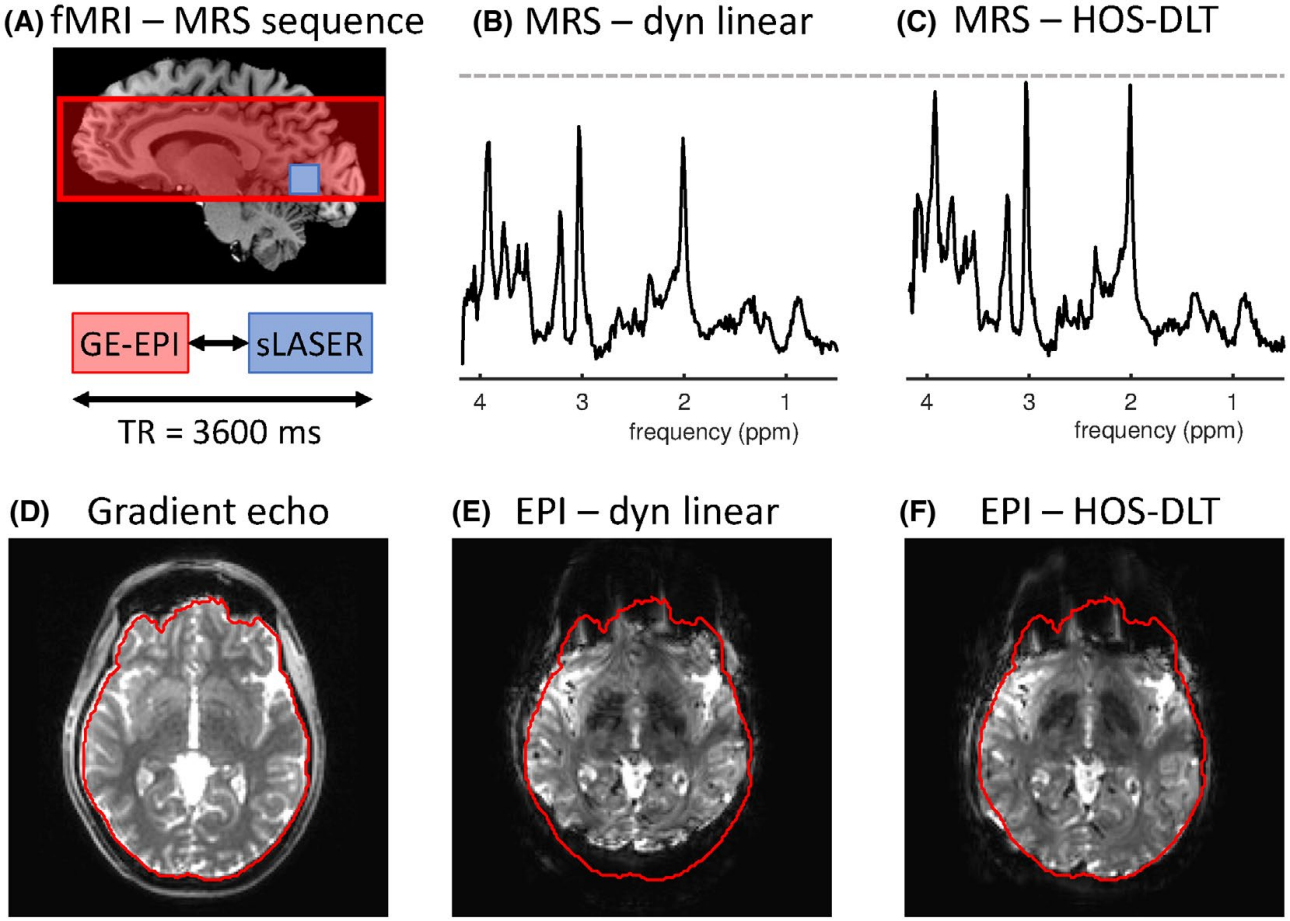
Interleaved MRS‐fMRI (A) can be performed by using dynamic linear shims. Although this would be an obvious approach up to 3T, higher order shimming is required at higher field. The MRS linewidth was improved using the higher order shims from 16 Hz linewidth using dynamic linear shimming (B) to 12 Hz linewidth with HOS‐DLT (C). At the same time, image distortions in the EPI are reduced. Compared to the gradient echo reference image (D) EPI imaging with linear shims leads to high image distortions at ultra‐high field (E). Using the third order HOS‐DLT shim (F) the EPI distortions are reduced while simultaneously enabling the use of higher order shimming for interleaved MRS

The single voxel sLASER MRS sequence was localized in the occipital cortex, where typically visual functionality is investigated, and was interleaved with a GE‐EPI imaging sequence, as typically used for fMRI.^28^ Because the focus of this work is image and spectral quality due to achieved B_0_ homogeneity, no visual stimulation was applied and functional analysis was omitted. A full GE‐EPI volume was interleaved within every TR of the MRS sequence (Figure 1A). Both the MRS and GE‐EPI had one start‐up dummy to reach steady state. The single‐shot GE‐EPI sequence had 35 slices with thickness of 2 mm, FOV of 200 × 232 mm^2^, in‐plane voxel size of 2 × 2 mm^2^, TE = 25 ms, and a SENSE^30^ factor of 3 was used. A short‐TE, non‐EPI gradient echo volume, with the same FOV and resolution was acquired as geometrical reference for comparison of distortions with different shim optimizations. A dynamic linear shim without setting the higher order shims was compared to third order HOS‐DLT, as such a dynamic linear shim would be an obvious choice for field strengths of 3T and lower.

### 2.10 MR motion navigated SV‐MRS

For single voxel MR spectroscopy with motion correction, the same sLASER sequence as described previously was used. For motion navigation, a fat‐selective 3D gradient echo sequence^31^ was inserted in every MRS‐TR, with an isotropic voxel size of 7 mm^3^, FOV of 256 × 256 × 161 mm^3^, TE/TR = 1.5/10 ms, binomial fat‐selective excitation with a flip angle of 1 degree, and a SENSE factor of 3 in both phase encoding directions. Motion updates were estimated and applied in real‐time using the iMOCO framework.^12^ A slice selective frequency measurement through the MRS voxel was used to track and update the zero‐order B_0_ field component at every TR of the MRS sequence.

In one subject, selectivity of the fat‐navigator and spectral linewidth were determined when using the MRS shim, whole brain shim (Equation 1), and the HOS‐DLT shim. Fat‐selectivity was visually assessed over the whole head. In another subject, motion correction performance was assessed when using the HOS‐DLT shim, by experiments with controlled movement, with and without motion correction. Visual instructions were given to the subject on how to perform the controlled movement during the experiments. The voxel was placed in the left frontal white matter, and the subject was instructed to move the head left, such that after motion, in case of no correction, the voxel would be localized in the medial frontal grey matter. With this motion paradigm, a large contrast between the grey and white matter is expected in especially the glutamate signal.^32^ Partial reconstructions of the MRS data using the first and last 6 acquisitions were performed to measure metabolite concentrations before and after motion with and without motion correction. This way the changes in metabolite concentrations due to the altered gray matter/white matter ratio in the voxel caused by motion could be quantitatively assessed.

The MRS data was analyzed using LCModel^33^ using a basis set including 20 metabolites – alanine, ascorbate, aspartate, creatine (Cr), gamma‐Aminobutyric acid, glutamine (Glu), glutamate, glycine, glycerophosphocholine (GPC), glutathione, myo‐Inositol (mIno), lactate, N‐acetylaspartate (NAA), N‐acetylaspartate‐glutamate (NAAG), phosphorylcholine (PCh), phosphocreatine (PCr), phosphorylethanolamine, scyllo‐Inositol, serine, and taurine – and a measured macromolecular baseline. Data quality was assessed on SNR on the highest peak in the spectra, Cramér–Rao lower bound (CRLB), and FWHM linewidth.

## 3 RESULTS

### 3.1 Field simulations in a group of subjects

**Figure 2:**
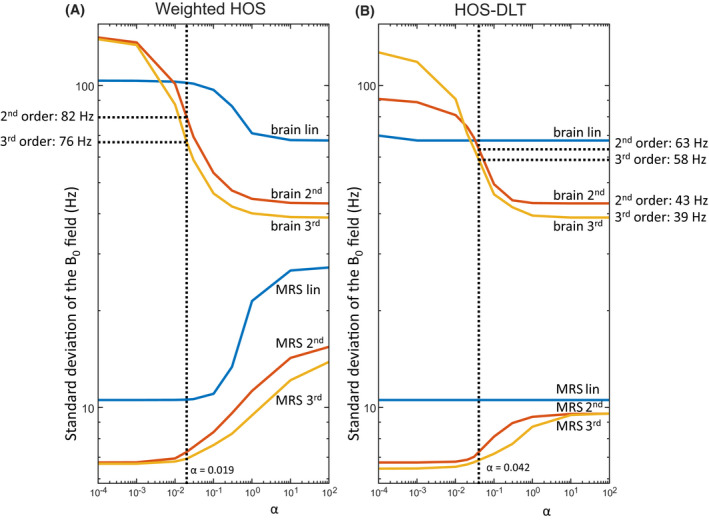
Analysis of the cost function weight for a single voxel MRS shim volume of interest (VOI) in the hippocampus in a single subject. Results for different shim orders are plotted on a double logarithmic scale as standard deviation over the respective shim VOI. With weighted static higher order shimming (A, wHOS), the α dependent trade‐off between brain and voxel homogeneity can be seen from the low standard deviation of the field with higher α (top three lines; blue for linear, red for second order and yellow for third order shimming) and the low standard deviation of the field in the voxel with low α (bottom three lines). At a cut‐off of 0.5 Hz above optimal MRS‐voxel shim for third order shimming at α = 0.019, the whole brain standard deviation reached 82 Hz and 76 Hz for second and third order respectively. Using weighted static higher order shimming with dynamic linear terms (B, HOS‐DLT), a higher α could be used (α = 0.042) to achieve a better whole brain homogeneity at similar MRS homogeneity. For linear shimming, optimal shimming was possible in all cases, for second order shimming 63 Hz and for third order shimming 58 Hz was reached. This is still above the optimal 43 Hz and 39 Hz that was reached with a whole brain optimization (or using a high alpha value) using second and third order shim order respectively

With both the wHOS shim and HOS‐DLT shim lower values of α give highest weight to the voxel homogeneity, resulting in similar MRS VOI homogeneity as compared to an optimization taking only the MRS shim VOI into account according to Equation 1. For high values of α, more weight is given to the whole brain homogeneity (Figure 2), resulting in similar whole brain homogeneity as compared to an optimization taking only the brain shim VOI into account according to Equation 1. Using the wHOS shim in this voxel location for this subject, the optimal α was found to be 1.9 × 10^−2^ giving an average whole brain homogeneity of 82 Hz with second order shimming, and 76 Hz with third order shimming. Using the HOS‐DLT shim, the optimal α was found to be 4.2 × 10^−2^ giving a whole brain homogeneity of 63 Hz with second order shimming, and 58 Hz with third order shimming. The HOS‐DLT shim gained better whole brain homogeneity than the wHOS shim at similar voxel homogeneity levels. Using the optimal α, these accomplished whole brain homogeneity values were still systematically higher than the optimal whole brain homogeneity of 43 Hz and 39 Hz for second and third order shimming respectively, a situation that would be achievable with full higher order dynamic shimming capabilities.^3^

**Figure 3:**
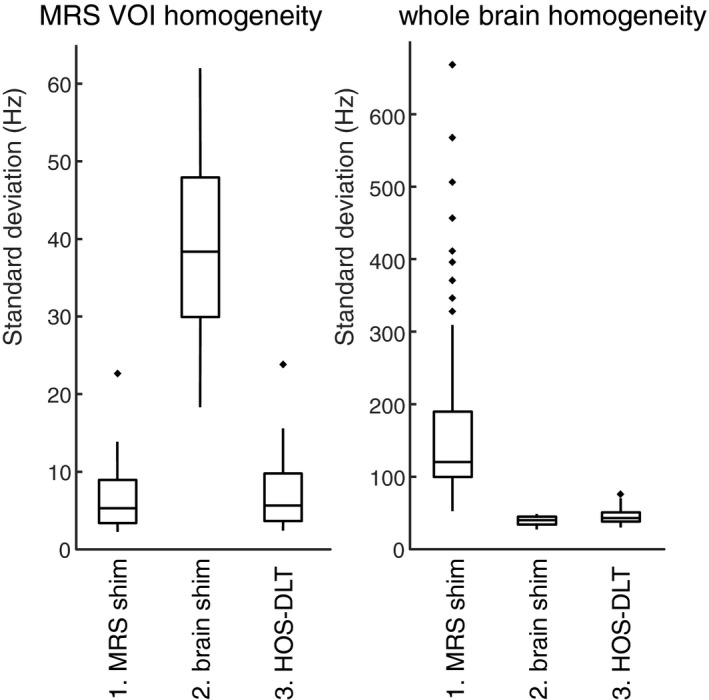
Box plots of the analysis for third order shimming summarized over 6 brain regions and 10 subjects. Good MRS volume of interest (VOI) shim homogeneity is traditionally achieved at the cost of a poor homogeneity in the whole brain shim VOI by using an optimization on the MRS VOI alone (1. MRS shim). With an optimization using only the brain shim VOI (2. brain shim) a good whole brain shim VOI homogeneity is achieved at the cost of a compromised MRS shim VOI homogeneity. Using the proposed weighted static higher order shimming with dynamic linear terms (3. HOS‐DLT) both a good voxel homogeneity and a good (but slightly compromised as compared to 2) whole brain homogeneity was reached

In Figure 3, the MRS shim VOI field homogeneity and whole brain field homogeneity are plotted over the group and brain regions, using third order shimming and the α value determined above. The MRS shim gives the lowest standard deviation of the field homogeneity in the shim VOI of 6.4 ± 3.9 Hz (mean ± std), at the cost of high whole brain homogeneity of 175 ± 132 Hz. The whole brain shim, on the contrary, gives the best whole brain homogeneity of 38.9 ± 6.0 Hz, but at significantly worse MRS shim VOI homogeneity of 38.9 ± 11.2 Hz. The HOS‐DLT shim shows marginally decreased local homogeneity in the MRS shim VOI of 7.0 ± 4.3 Hz as compared to the optimization on the MRS shim VOI alone and whole brain homogeneity of 45.3 ± 10.6 Hz, which is close to the best achieved whole brain homogeneity with the optimization on the brain alone.

### 3.2 Residual water suppression imaging

**Figure 4:**
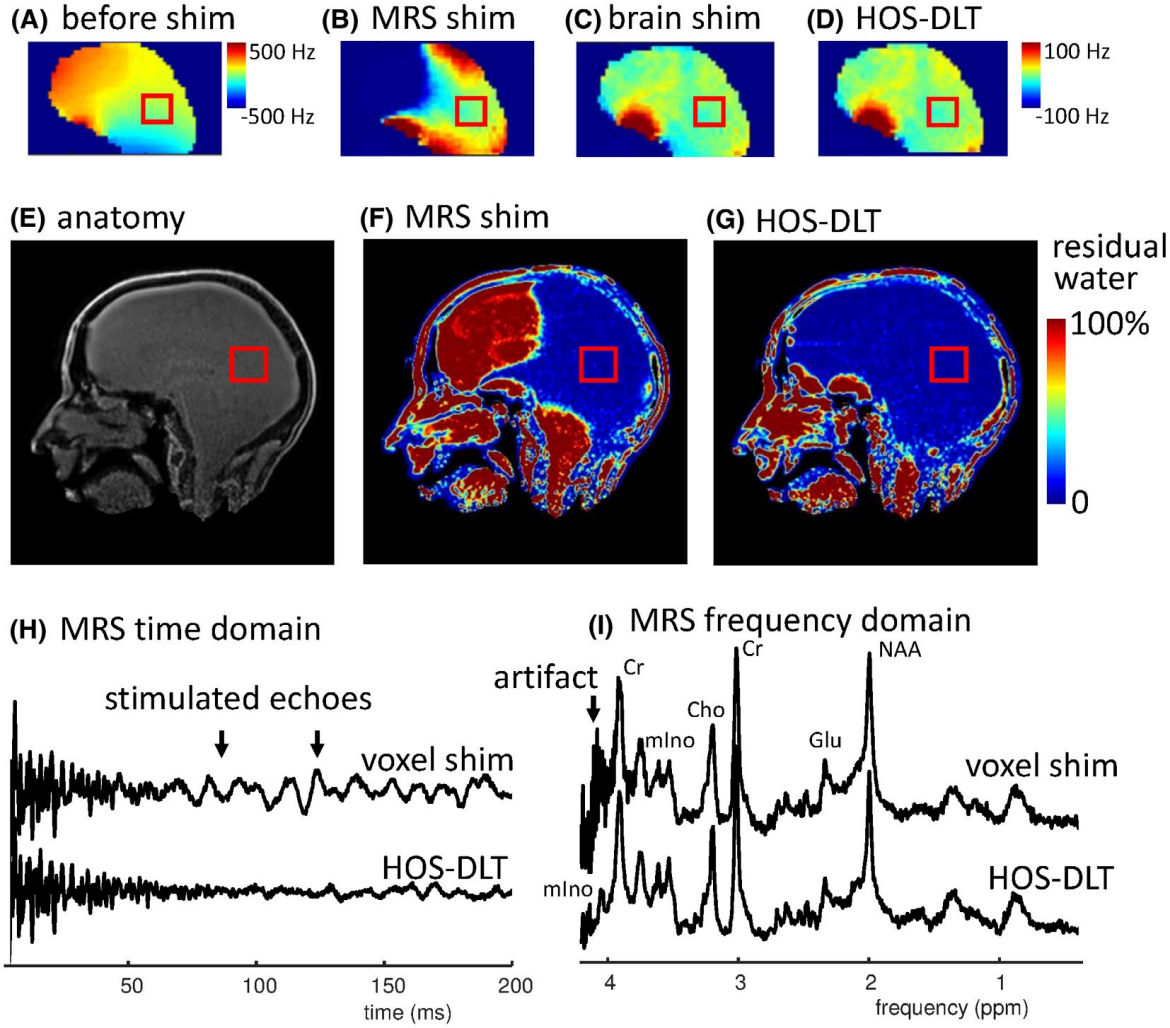
(A)‐(D) show B_0_ maps for different shimming approaches with a voxel in the occipital cortex. The best MRS shim volume of interest (VOI) homogeneity is reached with the localized MRS shim (B), however as a result, several parts of the brain are far off‐resonance. With a whole brain shim (C), the global field homogeneity is improved, at the cost of reduced homogeneity in the voxel. The achieved whole brain homogeneity that is used during water suppression when using HOS‐DLT is displayed in (D), during the MRS acquisition the linear shims are adjusted. The HOS‐DLT shim gives both good MRS VOI and whole brain homogeneity (note that B, C, D have the same scaling). (E) is the image without water suppression, used as denominator when calculating the water suppression ratio maps (F‐G). The result of large off‐resonance signals is seen in (F) where the water signal is not suppressed in large regions of the brain. With the HOS‐DLT shim, the extent of the water suppression is increased (G), reducing chance of stimulated echoes in the MRS data. An example of reduction of artificial signal from a poorly suppressed region is shown in time (H) and frequency (I) domain, where the 4.05 resonance of myo‐Inositol becomes visible only after suppression of the artifact

Residual water suppression imaging with the different shim approaches is shown in Figure 4. With a third order MRS shim several parts of the brain are so far off‐resonance that the water suppression outside the MRS voxel fails, leading to a high residual water signal in some off‐resonance areas, and a chance for generating stimulated echoes.^6^ With the third order HOS‐DLT shim, the water suppressed area is increased and chances for generation of stimulated echoes from outside of the MRS voxel are reduced while maintaining a similar linewidth for MRS. Figure 4 shows an MRS acquisition where a stimulated echo artifact^6^ was encountered when using the MRS shim VOI only. This was reduced using the HOS‐DLT shim, showing a better spectral quality around the 4.0 ppm region. This is indicative of a better water suppression in problematic areas in the head but outside of the MRS voxel.

### 3.3 Interleaved MRS‐fMRI

Figure 1 displays the image distortions encountered in the interleaved GE‐EPI images using dynamic linear shimming and the HOS‐DLT shim. Improved MRS quality was attained using the DLT‐HOS shim compared to the dynamic linear shim. The B_0_ field homogeneity in the MRS shim VOI improved from 3.8 Hz standard deviation for linear shimming to 2.4 Hz standard deviation of the field for HOS‐DLT. Measured linewidths from the voxel were 16 Hz for linear shimming vs 12 Hz for third‐order HOS‐DLT on creatine. At the same time, significant reductions in EPI image distortions are visible when going from dynamic linear shim to third order HOS‐DLT shim.

### 3.4 MR motion navigated SV‐MRS

**Figure 5:**
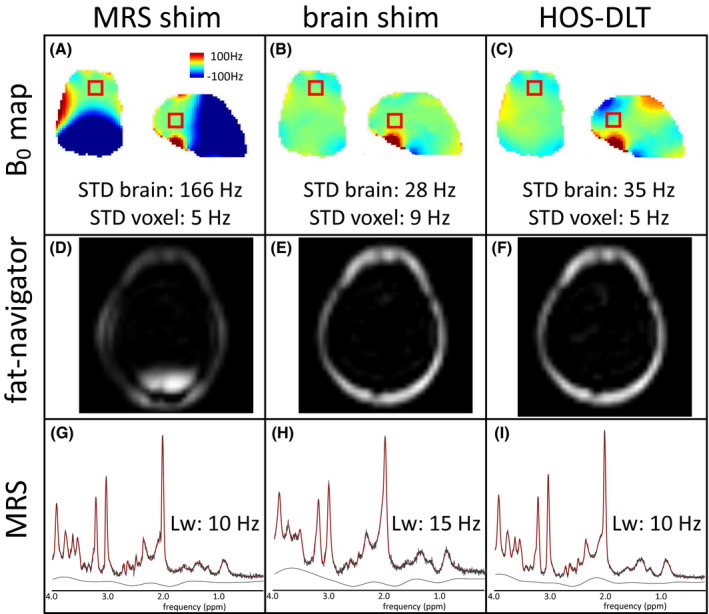
The B_0_ shim results (A‐C) in a single subject show the trade‐off between the optimization approaches for fat‐navigator interleaved MRS, with the fat‐navigator results shown in D‐F and the MRS results in G‐I. First, for the optimization on only the MRS shim volume of interest (VOI) (left column) we see a good homogeneity in the MRS voxel but high frequency offsets outside the MRS voxel (A). This compromises the fat‐selectivity of the navigator in the back of the head (D) but leads to good spectral resolution of 10 Hz (G). Second, a shim optimization on only the brain shim VOI (middle column) shows a good global homogeneity (B), resulting in good fat‐selectivity (E) but in reduced spectral resolution in MRS of 15 Hz (H). With the combined optimization using the HOS‐DLT shim (right column), both good global homogeneity was achieved (C, showing the HOS‐DLT map for the brain VOI shim) that allows for good fat‐selectivity (F) and good spectral resolution at 10 Hz was achieved (I)

The selectivity of the fat‐navigator, and the MRS linewidth are shown in Figure 5 for the MRS shim, the whole brain shim, and the HOS‐DLT shim. For this brain region, the third order MRS shim gave a shim VOI standard deviation of 5 Hz, resulting in a 10 Hz linewidth, and a whole brain standard deviation of 166 Hz, leading to a compromised fat‐selectivity in the posterior part of the head. The whole brain shim gave a standard deviation of 28 Hz over the whole brain and good fat‐selectivity, however at the cost of an increased field inhomogeneity in the MRS shim VOI to a standard deviation of 9 Hz, resulting in 15 Hz linewidth. The HOS‐DLT shim resulted in both a homogeneity in the MRS shim VOI with a low standard deviation of 5 Hz, and 10 Hz spectral linewidth, as well as a slightly compromised whole brain homogeneity of 35 Hz but still with good fat‐selectivity/water suppression over the whole brain.

**Figure 6:**
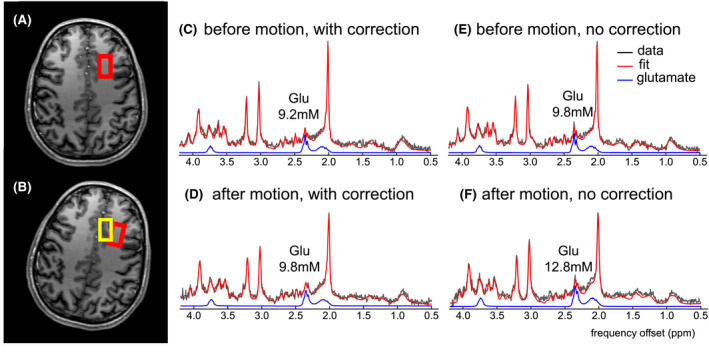
Real‐time corrected SV‐MRS is demonstrated using HOS‐DLT shimming with fat‐selective navigators. The subject was instructed to move halfway through a motion‐corrected SV‐MRS experiment in a controlled way. The MRS voxel was placed in the frontal white matter (A, indicated in red) but after motion without correction the voxel was located in the frontal gray matter (B, indicated in yellow), with higher expected glutamate levels. With a correct motion update, the voxel should remain located in the white matter. Segmented reconstructions from the acquisitions before motion (C,E) and after motion with correction (D) show similar glutamate levels, indicating that the motion correction is able to keep the voxel in white matter. After motion without correction, an increase in glutamate is seen (F), indicative of the displacement of the voxel into the gray matter

Figure 6 shows results of an MRS acquisition in frontal white matter with and without motion correction using the HOS‐DLT shim. When motion correction is not applied, the voxel covered an area containing a larger fraction of gray matter after motion than before motion. From the MRS data, an increase in estimated glutamate concentration was observed, indicating that the voxel shift from white to gray matter was left uncompensated. After the instructed movement with correction applied, the voxel is expected to remain in white matter. From the MRS data, similar levels of glutamate were measured on the partially reconstructed data, indicating that the motion correction correctly shifted the voxel to the new location.

Although the data obtained before motion, and after motion with motion correction, are visually of similar quality, the linewidth deteriorated slightly after motion from 15.2 Hz to 17.3 Hz on creatine and the SNR decreased from 26 to 21 after motion. CRLBs were the same before and after motion with motion correction (NAA+NAAG 2%, Cr+PCr 3%, GPC+PCh 4%, Glu 6%, mIno 5%).

## 4 DISCUSSION

An algorithm for optimizing both local and global shimming was developed, using a fixed set of higher order shims with dynamically varying linear terms (HOS‐DLT). This can be applied in high field and ultra‐high field MR systems equipped with higher order shimming coils, in sequences where both local and global homogeneity is important, but a full dynamic higher order eddy current compensation is not available.

Both the investigated static weighted higher order shimming (wHOS) and weighted higher order shimming with dynamic linear terms (HOS‐DLT) resulted in a trade‐off between global and local homogeneity. The trade‐off can be set by scaling the weighting parameter α, so that for low α the local homogeneity in the MRS shim VOI is favored, whereas for high α the global homogeneity for whole brain shimming is favored. We observed that by using dynamic linear terms, for similar homogeneity in the MRS VOI larger values of α could be used as compared to wHOS, resulting in a better trade‐off between local and global homogeneity. It should be noted, however, that the improvement between wHOS and HOS‐DLT is expected to be dependent on subject anatomy, voxel location, and shim order. The wHOS method is simpler to implement as only a single set of linear shims needs to be considered.

With the chosen weighting parameter α for HOS‐DLT, similar MRS shim VOI homogeneity was reached compared to optimization on the MRS shim VOI alone (0.5 Hz increase over the enlarged VOI). This comes at a decrease in whole brain homogeneity as compared to a whole brain shim; however, sufficient homogeneity was achieved for the investigated applications. Alternative approaches could be pursued to derive an alternative weighting value when a different trade‐off is desired. We expect this reoptimization to be required especially in cases with large deviating spatial distribution of magnetic susceptibility, such as with metallic implants or high iron deposition. Also a reoptimization of α might be needed for deviating MRS voxel locations or imaging volumes.

Notably, the weighting parameter was optimized on a single subject and a single central brain region (hippocampus). A gain could be found in assessing an appropriate weighting value for a specific brain region and/or on a subject basis. Although a single shim calculation only takes a short time (in the order of seconds for third order HOS‐DLT on the MRI console), running an optimization curve to find an optimal α could take a more significant amount of time; therefore, we chose here to use a single overall weighting parameter for all subjects and regions.

In this work the minimization target in the numerical simulations was chosen as the standard deviation in the respective shim ROI corresponding to the MRS voxel or the whole brain. Alternative approaches could be to use min‐max minimization or alternative histogram analysis values, tailored to the required shim field characteristics.

The proposed single step optimization would be preferred over iterative shimming, setting first only the higher order shims and second optimizing the linear shims for multiple target VOIs,^34^ although an iterative approach of HOS‐DLT with a secondary linear shimming could provide a simple compensation of possible higher order cross‐terms.^17^

An improvement was seen using third order shimming over second order shimming, and larger improvement of the HOS‐DLT approach is expected from shim systems with even higher order spherical harmonic coils^35^ without the need for eddy current compensation on higher order shim coils. Full higher order dynamic shimming could offer simultaneously the best voxel and whole brain shim; however, due to the required eddy current compensation the shim performance is also compromised leading to suboptimal results compared to the theoretical optimum.^20^ Alternatively, novel shimming approaches such as multicoil shimming could provide full dynamic shimming capabilities without the need of eddy current compensation.^22^ Finally, it is important to note that, apart from the accurate B_0_ shimming and the resulting linewidth decrease, several other factors contribute to quantification accuracy, such as residual water signal and out‐of‐voxel artifacts.^2^, ^6^

Three applications of the HOS‐DLT shim approach for improved global and local homogeneity were shown for single voxel MR spectroscopy at 7T. First, improved global shimming led to an improved overall water suppression, reducing the chances of detrimental stimulated echoes in single‐voxel MRS. The risk of stimulated echoes increases with the order of shimming and with field strength, as the out‐of‐voxel water frequencies are further off‐resonance. The larger water suppression region with the HOS‐DLT shim compared to the MRS shim reduces the risk of generating such stimulated echoes. Furthermore, the improved overall water suppression can help to better guide retrospective signal processing for removal of spurious echoes^29^ as possible artifact locations in the brain can be more easily identified.

Second, the HOS‐DLT shim enabled the use of higher order shimming in interleaved MRS‐fMRI, such that this technique could be used to assess neurochemical and hemodynamic responses. The HOS‐DLT shim showed improved MRS data quality and reduced EPI distortions compared to the dynamic linear shim, which would be an obvious choice at 3T. Although not tested here, both the EPI and MRS sequences can be run with an MRS VOI shim, resulting in good MRS linewidth but would usually be expected to result in extreme EPI distortions. Although post‐processing distortion correction^36^ can improve distortions to some degree, signal dropouts due to through plane dephasing are not retrievable.

Third, the HOS‐DLT optimization enabled the use of higher order shimming in navigator‐based, motion‐corrected single‐voxel MRS at 7T. Fat‐selective navigators were used for MRI‐based motion navigation. Due to both the prolonged T_1_ relaxation times and increased susceptibility distortions at 7T, the parallel‐imaging accelerated, non‐EPI, Cartesian fat‐navigators were shown to perform well for motion navigation. Fat‐navigators exhibited minimal MRS signal spoiling and negligible geometric distortions in the navigator images. However, fat‐selective motion navigators require global field homogeneity for the fat‐selective excitation, where poor fat‐selectivity leads to poor undersampling performance and a variable poor fat‐selectivity results in registration errors and corrupt motion estimates. Alternative motion correction approaches using either EPI‐navigators^11^, ^12^, ^37^ or spiral readouts^38^ are expected to similarly benefit from the whole brain improvements in field homogeneity provided by the HOS‐DLT shim, also at 3T. Motion‐corrected single‐voxel MRS utilizing HOS‐DLT shimming resulted in high data quality, similar to data acquired without motion. This is especially relevant for studies where children or severely ill patients are examined, as the risk of motion is increased in these groups. We did observe an increase in linewidth after motion despite performing motion correction, which was likely due to motion‐induced shim changes. Therefore, a potential next step would be to investigate dynamic shim updating in response to motion‐induced shim changes.^10^, ^39^

## 5 CONCLUSION

An algorithm for dynamic global and local shimming was presented using a fixed set of higher order shims and dynamic linear terms (HOS‐DLT). This enabled the use of higher order shimming on single voxel MRS, combined with sequence elements that require whole brain field homogeneity such as water suppression, interleaved EPI, and MR‐navigator based motion correction.

## CONFLICT OF INTEREST

Mads Andersen is an employee of Philips Healthcare.
